# sCLEC-2 (Soluble C-Type Lectin-like Receptor 2) as a New Diagnostic Marker of Platelet Activation in Colorectal Cancer Patients—A Preliminary Study

**DOI:** 10.3390/diagnostics16071004

**Published:** 2026-03-26

**Authors:** Violetta Dymicka-Piekarska, Mariusz Gryko, Anna Justyna Milewska, Blanka Wolszczak-Biedrzycka, Maja Aleksandra Oksentowicz, Elżbieta Motybel-Iwańczuk, Paweł Pawlak, Justyna Dorf

**Affiliations:** 1 Department of Clinical Laboratory Diagnostics, Medical University of Bialystok, Waszyngtona 15a St., 15-269 Białystok, Poland; 2 1st Department of General and Endocrine Surgery, Medical University of Bialystok, M. Skłodowskiej-Curie 24a St, 15-276 Białystok, Poland; 3 Department of Biostatistics and Medical Informatics, Medical University of Bialystok, Szpitalna 37, 15-295 Białystok, Poland; 4 Department of Psychology and Sociology of Health and Public Health, University of Warmia and Mazury in Olsztyn, Warszawska 30, 10-082 Olsztyn, Poland

**Keywords:** CLEC-2, colorectal cancer, platelets, inflammation, biomarker

## Abstract

**Background/Objectives**: CLEC-2 (C-type lectin-like receptor 2), the newest discovered platelet receptor, is involved in platelet activation and aggregation, the inflammatory response, tumor growth, metastasis, and angiogenesis. These unique features make CLEC-2 a promising candidate for a new biomarker and therapeutic target. The aim of our study was to evaluate the diagnostic value of CLEC-2 in patients with colorectal cancer (CRC). **Methods**: The serum CLEC-2 concentration was determined using ELISA methods in 64 CRC patients and 25 healthy subjects. **Results**: Our results indicate that the concentration of the serum CLEC-2 was significantly higher in CRC patients than in healthy subjects. Furthermore, the CLEC-2 levels were significantly higher in G3- than G2-grade CRC, and in patients with more advanced CRC, such as those with lymph node and distant metastases, than in patients without any metastases. CLEC-2 showed a positive correlation with platelet indices (PLT and MPV). The area under the ROC curve confirmed CLEC-2’s excellent diagnostic power in distinguishing between patients with CRC. **Conclusions**: Our results indicate that CLEC-2 may be associated with CRC development and suggest that the identification of this biomarker could be useful for determining CRC progression.

## 1. Introduction

Blood platelets are among the main constituents of the tumor microenvironment actively involved in the formation of cancer metastases [1]. Platelets protect tumor cells from being destroyed by the immune system through forming aggregates with them (TCIPA). Paradoxically, they are a source of numerous pro-metastatic/tumorigenic growth factors (TGF-β, PDGF), pro-angiogenic factors (VEGF, PDGF, bFGF), metalloproteinases (MMP-1, -2, -3, -9 and -14), cytokines, and chemokines (e.g., IL-1β, CXCL1, CXCL4, CXCL12, CCL5) [2]. The interaction of platelets between tumor cells and their microenvironment is enabled by surface receptors, including GP IIb/IIIa, P-selectin, and the newly discovered CLEC-2 (C-type lectin-like receptor 2).

CLEC-2 was originally identified in immune cells [3,4] and subsequently as a platelet/megakaryocytes receptor [5]. CLEC-2 belongs to platelet receptors that induce the tyrosine kinase network of signaling [6]. CLEC-2 can be activated by two exogenous protein ligands—snake venom protein rhodocytin [7,8,9] and brown seaweed extract fucoidan [5]. In addition, endogenous protein ligand, podoplanin (PDPN, also known as Aggrus, gp38), can also bind to CLEC-2 and mediate platelet activation and aggregation [5,10,11]. Podoplanin is a small mucin-like protein expressed on a variety of cell types, including human kidney podocytes rat lung type I cells, macrophages [5,12], fibroblasts and osteoblasts [13], and some tumor cells, such as brain and breast cancer, and squamous carcinoma [12,14,15,16].

Similarly to elevated platelet counts, high podoplanin expression may be associated with an increased incidence of cancer metastasis to lymph nodes [4]. Platelet interactions with tumor cells via the podoplanin-CLEC-2 complex contribute to tumor cell invasion and metastasis by platelet aggregation and protection of tumor cells against the immune system [4,17]. Recent studies have shown that although the role of CLEC-2 is uncertain, it may be a new potential therapeutic target [12,15,18], as blocking it by PDPN antibodies inhibits tumor metastasis, platelet aggregation, and thrombosis formation in mice [19,20].

CLEC-2 is a surface receptor which is also secreted as a protein that can be measured in serum or plasma in a soluble form (sCLEC-2). To our knowledge, this is one of the few studies carried out to examine the diagnostic significance of CLEC-2 as a circulating platelet-associated biomarker in patients with colorectal cancer (CRC). We chose to address this topic because previous, limited studies on the use of CLEC-2 as a biomarker produced inconclusive results.

The aim of this study was to evaluate the diagnostic significance of soluble C-type lectin-like receptor 2 (sCLEC-2) as a circulating platelet-related biomarker in patients with colorectal cancer (CRC). Specifically, we sought to determine whether serum sCLEC-2 levels are associated with platelet activation markers, angiogenic factors, and clinicopathological features of CRC, as well as to assess its potential usefulness in distinguishing CRC patients from healthy individuals.

## 2. Material and Methods

### 2.1. Study Group

The study was conducted in accordance with the Declaration of Helsinki and approved by the Bioethics Committee of Medical University of Bialystok, Poland, permission number APK.002.21.2020. All participants gave their written consent to participate in the study involving humans and were informed about the purpose of the study.

The study was conducted on 64 patients with primary colorectal cancer (Adenocarcinoma), 20 women and 44 men, of average age of 65 years, qualified for surgical resection of the tumor in 2020–2022. The patients were diagnosed based on physical examinations, imaging tests (CT), and colonoscopy at the 2nd Department of General and Gastroenterological Surgery of the University Hospital in Bialystok. The patients were divided depending on the stage of advancement according to the TNM classification (AJCC) into four groups: I stage (T_1–2_ N_0_M_0_), II stage (T_3–4_ N_0_M_0_), III stage (T_1–4_ N_1–2_M_0_), and IV stage (T_1–4_N_1–2_M_1_).

More often, the tumor was located on the left side of the colon (61%), while it was located on the right side of the colon in 39% of cases. Over 72% of patients had tumor size of over 30 mm. The percentage of patients without lymph node (N0) and distant metastasis (M0) was 61% and 91%, respectively. The tumor differentiation grade (G) was G2 (moderately differentiated) in 58 patients and G3 (poorly differentiated) in six patients. Detailed, clinical characteristics of the study group are presented in Table 1.

The exclusion criteria in patients with CRC included systemic inflammatory, autoimmune, and infectious diseases. Additionally, those who had taken oral anticoagulants, anti-inflammatory, steroid, and antiplatelet drugs in the last 2 weeks were excluded from the study.

The control group consisted of 25 healthy subjects, who were age- and gender-matched, and who were undergoing their periodical check-up at the Medical University Hospital Clinic. The same exclusion criteria were used in control group as in the study group.

### 2.2. Material

Venous blood samples (2.6 mL) were collected into serum tubes (S-Monovette, SARSTEDT, Nümbrecht, Germany) and processed according to a standardized protocol to minimize pre-analytical variability. All samples were allowed to clot at room temperature, and they were centrifuged within 30 min of collection for 15 min at 1000× *g* to obtain serum. Immediately after centrifugation, serum was aliquoted to avoid repeated freeze–thaw handling and stored at −80 °C until analysis. Each sample underwent only one freeze–thaw cycle prior to the ELISA assays.

The duration of storage at −80 °C did not exceed the maximum period recommended by the manufacturer for preserving protein stability. Before measurement, the samples were thawed gradually on ice and mixed gently using a vortex to ensure homogeneity without inducing foam formation or protein degradation.

### 2.3. CLEC-2 and VEGF Concentration

Serum concentrations of CLEC-2 and VEGF were determined using commercially available ELISA kits (Human CLEC-2 ELISA Kit, RayBiotech, Parkway Lane, Suite 200 Peachtree Corners, GA, USA; Human VEGF Quantikine ELISA Kit, R&D Systems, 19 Barton Lane, Abingdon Science Park, Abingdon, UK), following the manufacturers’ instructions. The intra-assay and inter-assay coefficients of variation declared by the manufacturers were within acceptable analytical ranges for research applications. All samples were tested in duplicate, and no significant differences between replicate measurements were observed. The minimum detectable concentrations (MDD) for CLEC-2 and VEGF were 1.22 ng/mL and <5 pg/mL, respectively.

These procedures ensured controlled pre-analytical conditions and analytical reliability; however, comprehensive assay validation (e.g., repeated measurements across multiple days, assessment of lot-to-lot variability) was beyond the scope of this preliminary study.

### 2.4. Platelets

PLT (platelet count) and MPV (mean platelet volume) were automatically determined on the hematological analyzer XN-1000 (Sysmex, Hamburg, Germany). It is a fully automated five-part differential hematology analyzer.

#### Statistical Analysis

In the statistical analysis, the normality of the distribution of quantitative variables was verified using the Shapiro–Wilk test. Because the distribution of the analyzed variables was not normal, non-parametric tests were used for the comparisons: the Mann–Whitney U test for two groups and the non-parametric Kruskal–Wallis rank-order ANOVA, with a post hoc multiple comparison of mean ranks for all samples in the case of multiple groups. The Spearman rank-order correlation coefficient was also calculated, and its statistical significance was assessed. In addition, linear regression analysis was performed to evaluate the relationship between selected quantitative variables and to explore their independent contributions to the outcomes.

The receiver operating characteristic (ROC) analysis was conducted to evaluate the predictive performance of CLEC-2, CEA, CA19-9, and their combined model (CLEC-2+CEA, CLEC-2+CA19-9). To formally compare the diagnostic accuracy of the ROC curves, the DeLong test was applied, and the corresponding *p*-values were reported. The predictive strength of the models was expressed using the area under the ROC curve (AUC) and odds ratios (ORs) with 95% confidence intervals.

Following methodological recommendations, extensive internal validation using non-parametric bootstrap resampling (1000 replicates) was carried out. Bootstrap procedures were used to estimate optimism-corrected AUC values and to validate the stability of odds ratios for the CLEC-2+CEA model. Post hoc statistical power analysis was performed for the primary endpoint, as well as for the bootstrap-validated AUC and OR estimates.

Statistical significance was set at *p* < 0.05. All classical statistical analyses were performed using the Statistica 13 software package (TIBCO Software Inc., Palo Alto, CA, USA (2017),) and GraphPad Prism 9.5.1 (GraphPad Software, Inc., San Diego, CA, USA). Bootstrap validation, linear regression modeling, and power analyses were conducted in Python 3.10 using custom scripts.

## 3. Results

### 3.1. CLEC-2 Concentration

Serum concentration of CLEC-2 was significantly higher in CRC than in control group without cancer (*p* < 0.0001) (Table 2). The CLEC-2 concentration was significantly higher in G3 tumor than in G2 tumor (differentiation grade *p* < 0.0001). The CLEC-2 concentration indicates growing tendency with TNM classification of CRC, as significant differences were observed between stage I and IV (*p* = 0.01). CLEC-2 does not significantly differ between the patients with or without lymph node and distant metastasis, depth of tumor invasion (pT), histological type, and/or CRC localization and size of CRC (Table 2).

### 3.2. VEGF Concentration

The VEGF concentration was also significantly higher in the CRC patients as compared to the control group (*p* < 0.001) (Table 2). The statistical analysis indicated that the patients with lymph node and distant metastasis had a significantly higher VEGF concentration than the patients without any metastasis (*p* = 0.050, *p* = 0.005, respectively). We observed statistically significant differences in VEGF concentration between CRC stage I and IV (*p* = 0.0471) and between stage II and IV (*p* = 0.0258). VEGF does not significantly differ between CRC localizations, tumor size, histological type, and depth of tumor invasion (pT) of CRC (Table 2).

### 3.3. Platelet Indices

The statistical analysis indicated that the PLT count and MPV were significantly higher in the CRC patients as compared to the control group (*p* = 0.001, *p* < 0.0001, respectively) (Table 2). The PLT count was also higher in patients with lymph node and distant metastasis (*p* = 0.01, *p* = 0.002, respectively) (Table 2).

### 3.4. Spearman Correlations

**Figure 1:**
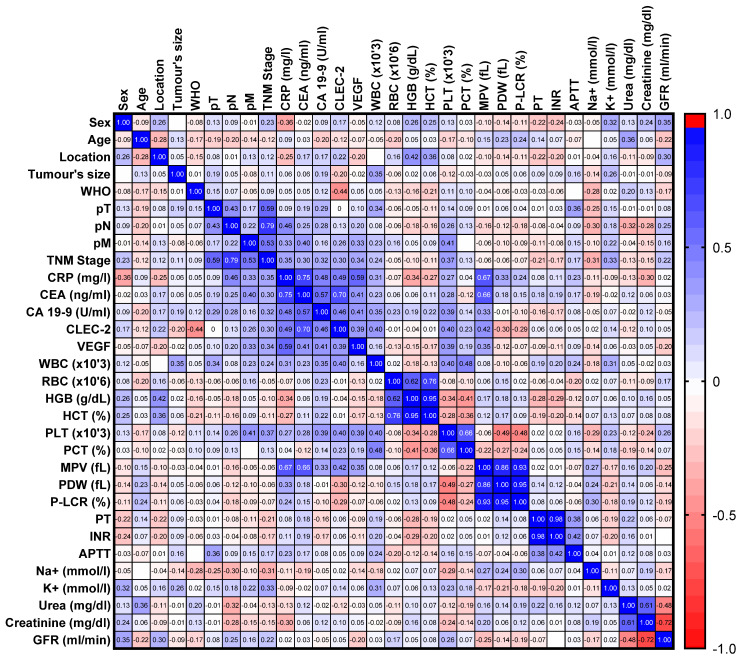
The heat map of the correlation of selected parameters in colorectal cancer (CRC) patients.

In the CRC patients, we observed a positive correlation of CLEC-2 with the PLT count (R = 0.398, *p* = 0.006), MPV (R = 0.424, *p* = 0.003), and VEGF (R = 0.3875, *p* = 0.007) (Figure 1). We also demonstrated positive correlations between CLEC-2 and CRP (R = 0.495, *p* = 0.003) and tumor markers: CEA (R = 0.703, *p* < 0.0001) and CA19-9 (R = 0.464, *p* = 0.004) (Figure 1).

### 3.5. ROC Curve Analysis

**Figure 2:**
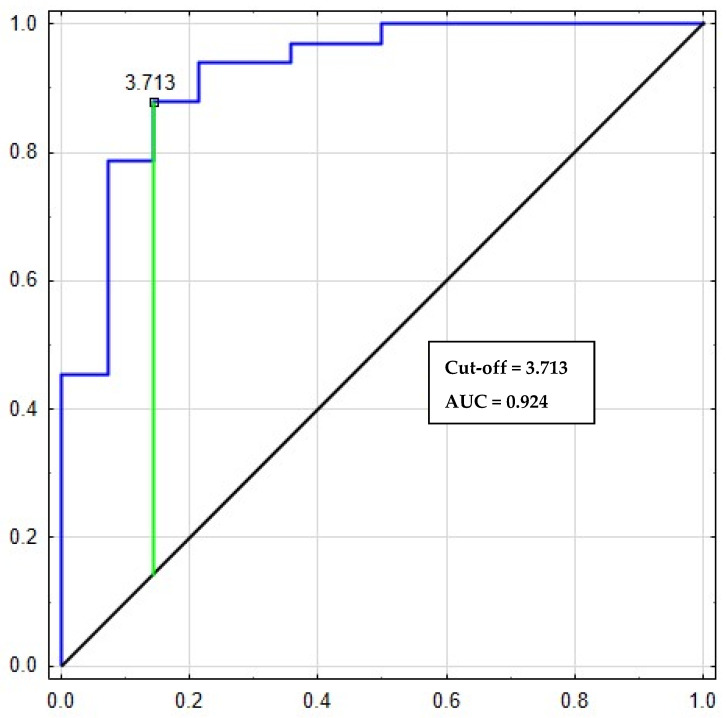
Receiving operating characteristic (ROC) curves of CLEC-2 for differentiating CRC patients with healthy subjects.

The relationship between diagnostic sensitivity and specificity was illustrated by a ROC curve (Table 3). It shows that areas under the ROC curve for CLEC-2 was 0.993, which indicates excellent diagnostic utility in differentiation between the CRC patients and the healthy subjects (Figure 2). At cut-off = 3.713 for CLEC-2, its sensitivity and specificity were 87% and 85%, respectively.

### 3.6. Comparison of CLEC-2 with Standard CRC Tumor Markers

To further evaluate the diagnostic performance of sCLEC-2, we compared its ROC curve with those of established tumor markers, CA19-9 and CEA, using the roccomp test and an algorithm suggested by DeLong, DeLong, and Clarke-Pearson.

**Figure 3:**
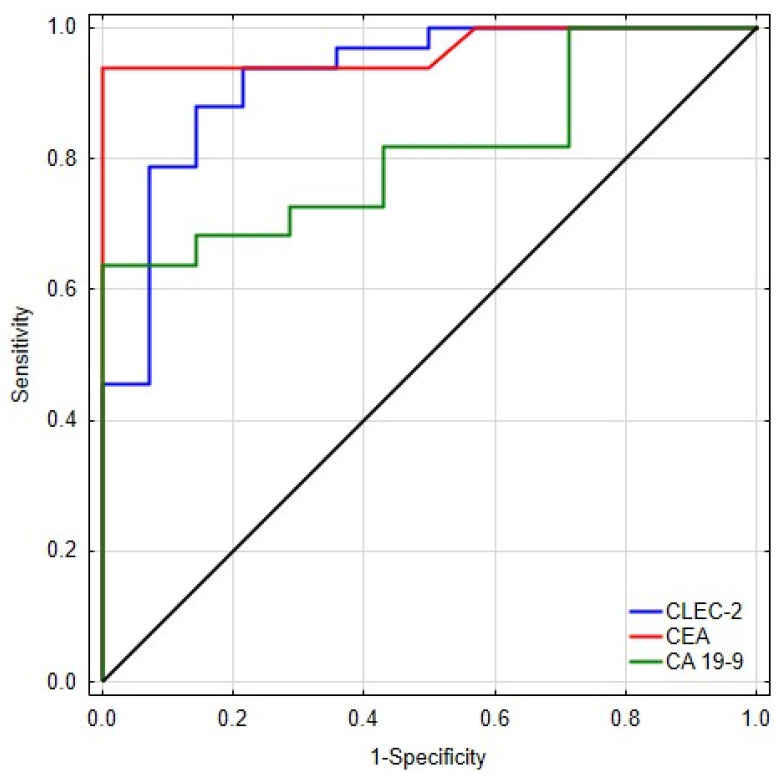
Receiver operating characteristic (ROC) curves for CLEC-2, CEA, and CA19-9.

Against CA19-9, sCLEC-2 demonstrated a higher AUC (0.938 vs. 0.812), although the difference did not reach statistical significance (χ^2^(1) = 2.36, *p* = 0.124). Similar findings were observed for the comparison with CEA, where the AUCs of sCLEC-2 and CEA were comparable (0.955 vs. 0.967; χ^2^(1) = 0.05, *p* = 0.827) (Figure 3) (Table 4).

Taken together, these results indicate that sCLEC-2 exhibits diagnostic performance that is at least comparable to and numerically higher than standard CRC biomarkers, although the differences did not achieve statistical significance, likely due to the modest sample size in each comparison subgroup. The consistently high AUC values (>0.93) across all analyses further support the potential diagnostic utility of sCLEC-2.

### 3.7. Bootstrap Validation

Bootstrap internal validation (1000 replications) for CLEC-2 confirmed high diagnostic power: AUC (apparent) = 0.924 (95% CI bootstrap: 0.827–0.987). AUC after optimism correction was 0.924. In univariate logistic regression, OR at 1 ng/mL = 6.36 (95% CI Wald: 2.09–19.37; 95% CI bootstrap: 2.96–313.54). The one-sided test H_0_: AUC = 0.90 was insignificant (*p* = 0.261), which, with a small number of observations (N = 89; 64 CRC, 25 controls) reflects a wide SE AUC. Detailed results, including the ROC curve and 95% CI bands, are presented in the Supplementary Materials (Figure S1, Table S1).

### 3.8. Logistic Regression

The univariate logistic regression analysis showed that five markers played a significant role in predicting CRC development, with all variables tested showing statistical significance. The results indicate complex relationships between increases in VEGF, CLEC-2, PLT, MPV, and WBC, as well as the probability of a diagnosis of colorectal cancer. The results of the analysis indicate that an increase in the VEGF concentration of 1 pg/mL is associated with a 4.181-fold increase in the odds ratio (95% CI: 1.865–9.375, *p* = 0.001). A regression coefficient, β, of 1.431 suggests a significantly positive logarithmic relationship between the VEGF levels and the probability of CRC. The highest odds ratio was obtained for the CLEC-2 receptor, where a 1 ng/mL increase in the concentration of this marker is associated with a 6.35-fold increase in odds (95% CI: 2.086–19.371, *p* = 0.001). The platelet analysis (PLT) revealed that each increase in the platelet count by 1000/µL was associated with an odds ratio of 1.011, which translates into a 1.1% increase in the odds of developing CRC (95% CI: 1.002–1.020, *p*-value: 0.015). In the case of MPV, the results indicated an OR of 3.941, suggesting that higher MPV values increase the odds of CRC by almost four times (95% CI: 1.870–8.304, *p*-value: <0.001). The last parameter examined was white blood cells (WBCs), for which the OR was 1.392. This means that each increase in white blood cell count by 1000 increased the chances of a positive result by 39.2% (95% CI: 1.008–1.921, *p*-value: 0.044) (Table 5).

## 4. Discussion

The binding between a tumor and inflammation cells is enabled due to functionally compatible receptors and molecules present on the cells’ surface. CLEC-2 shows increased expression on platelets and various inflammatory cells, and its physiological ligand, podoplanin (PDPN), is present on many neoplastic cells. So far, most of the studies have focused on the signaling and function of CLEC-2 on platelets.

We demonstrated that CLEC-2 can be cleaved from cell membranes and measured in serum or plasma in a soluble form. According to Etemad et al., binding PDPN to CLEC-2 leads to activation of platelets [21]. Our study revealed a sCLEC-2 concentration almost twice as high in the serum of CRC patients compared to healthy individuals, which indirectly suggests greater CLEC-2 expression on tumor-activated platelets. These findings align with and complement previous research exploring the biological role of CLEC-2 in cancer. Zhang et al. [22] demonstrated elevated sCLEC-2 levels in colorectal cancer, particularly in patients with liver metastases, suggesting that CLEC-2 may reflect tumor-associated platelet activation and metastatic potential. In contrast, Etemad et al. [21] reported decreased CLEC-2 expression on platelets and reduced plasma sCLEC-2 following platelet activation, which they attributed to receptor internalization. Although these observations may initially appear divergent, together they highlight the dynamic behavior of CLEC-2 in the tumor microenvironment. Our results, showing markedly increased sCLEC-2 concentrations in CRC patients and strong associations with advanced disease stages, support the hypothesis that CLEC-2 is shed from activated platelets and that it participates in interactions with tumor and inflammatory cells. When considered alongside the broader immunothrombotic functions of CLEC-2 described by Etemad [21] and colleagues and the metastatic associations reported by Zhang et al. [22], our study reinforces the concept that CLEC 2 represents a biologically relevant and clinically promising biomarker in colorectal cancer. Importantly, our study differs fundamentally from the work of Zhang [22] and Etemad [21], as we focused on soluble CLEC-2 measured directly in serum and its clinical associations in CRC, whereas their studies examined distinct biological contexts, Zhang [22] assessing sCLEC-2 mainly in relation to metastatic burden and Etemad [21] analyzing platelet-bound CLEC 2 and its behavior upon platelet activation.

What is more, we observed statistically significant differences depending on the degree of differentiation (G) of CRC and its clinical stage according to the TNM classification. Specifically, we observed significantly higher CLEC-2 concentrations in patients with poorly differentiated CRC and in those with stage IV disease (i.e., distant metastases to the liver and lymph nodes) compared to patients whose cancer was limited to the intestinal wall (stage I and II). Our results are in line with Zhang et al., who observed higher sCLEC-2 levels in CRC patients, especially those with liver metastasis [22]. In contrast, Etemad et al., showed that the CLEC-2 expression on platelets and the sCLEC-2 levels in plasma are decreased upon PLT activation. They hypothesized that it could be associated with the internalization of CLEC-2 [20]. We hypothesize that CLEC-2 could be shed from the platelet surface to form complexes with tumor and inflammatory cells. We also demonstrated that a higher platelet count (PLT) and greater mean platelet volume (MPV) had increased likelihood of being found in the CRC patients than in the control group—indicating the involvement of active platelets in this disease. PLT increased significantly in patients with metastases in lymph nodes and distant organs compared to patients without metastases. PLT also showed an upward trend with stage of advancement, as there were statistically significant differences between stages I and IV, thus confirming the involvement of PLT in the spread of cancer and formation of metastases. Numerous studies have shown that an increased platelet count is associated with advanced colorectal cancer and is an unfavorable prognostic marker [23]. MPV reflects platelet activity and is commonly used as its marker [24]. Physiologically, MPV is inversely proportional to PLT; therefore, increased platelet production is accompanied by a reduction in their mean volume. However, in many pathologies, such as cancers, this physiological ratio is disturbed, as demonstrated in our work.

In the regression analysis, CLEC-2 emerged as the strongest independent predictor among all evaluated parameters. The obtained odds ratio indicates that each 1 ng/mL increase in circulating CLEC-2 was associated with a 6.35-fold higher odds of colorectal cancer (95% CI: 2.086–19.371, *p* = 0.001). This substantial effect size suggests that even relatively small elevations in the CLEC-2 concentration may have meaningful diagnostic implications.

The correlation analysis revealed a moderate relationship between CLEC-2 and platelet indices—PLT and MPV—which confirms its relevance to platelet physiology. We also showed positive correlations between CLEC-2 and CRP and tumor markers, CEA and CA19-9, indicating that their elevation may reflect not only platelet-related changes but also broader inflammatory and tumor-associated processes.

Assessing the diagnostic power shows whether the study parameters can be used to diagnose and differentiate between healthy subjects and patients with colorectal cancer. Our ROC analysis revealed that CLEC-2 and MPV are our most reliable biomarkers of CRC, with high AUC values of 0.924 and 0.957, respectively. This confirms their excellent potential as new biomarkers associated with platelet activation.

Previous studies indicate the participation of CLEC-2 and its ligand not only in the development of cancer metastases but also its association with an increased risk of thrombotic complications (thromboinflammation) [25] and regulation of the immune response [11]. Experimental studies on mouse models indicate that CLEC-2 deficiency or blocking it with monoclonal antibodies may inhibit thrombus formation in tumor vessels and inhibit the formation of metastases. Simultaneously, an increase in tumor vascularisation was observed, thereby improving oxygen and nutrient supply, which indirectly promotes tumor proliferation and growth [17]. Antibody-mediated inhibition of this pro-aggregation protein on NF-17 cells reduced lung metastasis in vivo [17].

Recent studies also show that CLEC-2 may be a bridge between thrombosis and inflammation, which are closely related to cancer. As stated previously, CLEC-2 is strongly expressed not only on platelets and megakaryocytes but also on monocytes and macrophages, NK cells, and endothelial cells. What is more, Kerrigan et al. demonstrated that this receptor is also expressed by peripheral blood neutrophils [26]. According to their study, expression of CLEC-2 appears upregulated upon neutrophil migration from the bone marrow into the blood and downregulated again following the recruitment to a site of inflammation.

We described the involvement of chronic inflammation in the development of CRC in earlier numerous studies [27]. As has been clearly demonstrated, many cells involved in inflammation infiltrate neoplastic tissue, building tumor microenvironments [1,28] and regulating the balance between their pro- and anti-neoplastic activity [29]. In addition to platelets, leukocytes can also infiltrate the tumor microenvironment and can comprise up to 50% of the tumor mass. Interactions between tumor cells and macrophages further contribute to tumor progression by stimulating cytokine production. It has been demonstrated that CLEC-2 is one of the receptors regulating the inflammatory response by activating macrophages [30]. The regulatory role of CLEC-2 depends on the expression of its ligand, podoplanin, which is increased in inflammatory macrophages and independent of activation and release from platelets. It reduces the transport of macrophages from the tissues affected by the inflammatory process to the surrounding lymph nodes and inhibits the activation of macrophages, thus leading to a reduction in inflammation. Bourne et al. also showed that the treatment of inflammation with rCLEC-2-Fc induces rapid migration of inflammatory macrophages, which is associated with a simultaneous decrease in the proinflammatory cytokine TNF-alpha and an increase in the level of immunosuppressive IL-1 [11,30]. Increased recruitment and activation of macrophages drives tissue inflammation, which is observed not only in cardiovascular disease or infection but also in the course of neoplastic disease. Meanwhile, Kerrigan’s research indicated that CLEC-2 is expressed on the circulating neutrophils and can modulate inflammatory response, inducing cytokine production (e.g., TNF-alfa) in neutrophils [26]. While the treatment with rCLEC-2-Fc reduces TNF-α secretion and increases IL-10, CCL-2, CCL-5, and CXCL-1 in the inflammatory site, it also accelerates the removal of macrophages from the inflammatory site and induces T cell activation [30], which facilitates cancer cell recognition and destruction. It has been hypothesized that CLEC-2 is involved in the interaction between platelets and neutrophils because its ligand(s) are recognized on both [26]. This interaction could be associated with controlling infection, limiting inflammation, and/or suppressing disease development [31]. Due to its immunomodulatory role, CLEC-2 could be a novel target for reducing tissue inflammation, which can also take part in anti-cancer therapeutic strategies.

According to Takagi et al., the interaction between CLEC-2 and podoplanin on the surface of cancer cells promotes tumor growth, metastasis, and angiogenesis [32]. Following podoplanin binding, the hemITAM motif and Syk undergo phosphorylation by Src. Then, activated Syk phosphorylates hemITAM, Src, and a signaling complex consisting of LAT, SLP-76, and PLCγ2. Finally, PI3K engagement leads to platelet activation and the secretion of growth factors such as EGF, VEGF, PDGF, and TGF-β. VEGF, the most potent stimulator of angiogenesis, triggers new vessel formation in the primary tumor and at sites of distant metastases. VEGF also induces permeability of endothelial cells (ECs) and facilitates the extravasation of cancer cells [33]. The formation of new vessels within the tumor improves its oxygen and nutrient supply, indirectly promoting tumor proliferation and growth. Previous research on human and animal tumors indicates that VEGF is highly expressed in growing tumor tissues compared to healthy tissues [34].

We assessed the concentration of vascular endothelial growth factor (VEGF) in colorectal cancer (CRC) patients, as it is the main proangiogenic factor associated with platelets. Our study showed that angiogenesis is stimulated in CRC patients, reflected by a highly statistically significant increase in the VEGF concentration compared to healthy subjects. The VEGF levels differed significantly depending on the stage of clinical advancement of colorectal cancer. Furthermore, the VEGF levels were significantly higher in the patients with lymph node and distant metastases than in the patients without metastasis. Moreover, our study may indicate its involvement in the formation of metastases, thus spreading the cancer to both the lymph nodes and the liver. The correlation analysis revealed a moderate relationship between VEGF and PLT in CRC patients, which could suggest that VEGF originates from platelets.

In conclusion, our results demonstrated that soluble CLEC-2 detection may be an important biomarker associated with platelet activation in colorectal cancer and could be associated with cancer development and metastasis formation. This makes it an interesting target for preventing platelet aggregation and metastasis formation, but it needs to be studied further.

Several limitations of this study should be acknowledged. The analyses were conducted on a relatively small patient cohort, and certain subgroup evaluations; particularly those concerning G3 tumors, stage IV disease, or distant metastases were based on very limited sample sizes. This constraint not only reduces the statistical power but also restricts the possibility of performing more advanced and robust statistical analyses. Due to the relatively small cohort size and the limited number of cases in key clinicopathological subgroups, constructing multivariable models would risk unstable coefficient estimates, inflated standard errors, and overfitting. For this reason, multivariable logistic regression was not attempted. This represents an inherent limitation of the present preliminary analysis, and future research based on larger and more heterogeneous CRC populations will be necessary to enable robust multivariable modeling and to determine whether sCLEC-2 provides independent diagnostic value beyond established biomarkers such as CEA and CA19-9. Despite these limitations, a notable strength of the present study is the carefully selected cohort of CRC patients and the well-defined control group free of comorbidities. The current results may thus constitute an important preliminary foundation for subsequent clinical investigations aimed at assessing CLEC-2 and its diagnostic potential in broader colorectal cancer populations

Another important limitation of this study is its single-center design, which may restrict the generalizability of the findings to broader and more heterogeneous colorectal cancer populations. In addition, the cross-sectional nature of the study precludes assessment of temporal changes in sCLEC-2 levels, prognostic implications, or causal relationships between biomarker alterations and disease progression. Future multicenter, longitudinal studies will therefore be essential to validate the diagnostic utility of sCLEC-2 and to determine its potential role in patient monitoring or risk stratification.

## Figures and Tables

**Table 1:** Demographic and clinical data of colorectal cancer patients.

Variable	N (%)
**Age**	
<60 years	12 (19%)
≥60 years	52 (81%)
**Gender**	
Male	44 (69%)
Female	20 (31%)
**Location**	
Right side	25 (39%)
Left side	39 (61%)
**Tumor Size**	
<3 cm	18 (28%)
≥3 cm	46 (72%)
**Grade**	
G2	58 (91%)
G3	6 (9%)
**Histological Type**	
Adenocarcinoma	55 (86%)
Mucinous adenocarcinoma	9 (14%)
**Depth of Tumor Invasion (pT)**	
T1	0 (0%)
T2	16 (25%)
T3	41 (64%)
T4	6 (9%)
**Lymph Node Metastasis (pN)**	
N0	39 (61%)
N1 + 2	25 (39%)
**Distant Metastasis (pM)**	
M0	58 (91%)
M1	6 (9%)
**Stage at Diagnosis (TNM)**	
I	14 (22%)
II	23 (36%)
III	21 (33%)
IV	6 (9%)

**Table 2:** Platelet parameters results in colorectal cancer (CRC) patients in relation to the control group and depending on the clinicopathological characteristics of the tumor. Q1—25th percentile, Q3—75th percentile.

		CLEC-2 [mg/mL] Median (Q1–Q3)		PLT [×10^3^/µL] Median (Q1–Q3)		MPV [fl] Median (Q1–Q3)		VEGF [pg/mL] Median (Q1–Q3)	
**Group Tested**	CRC	5.33 (4.24–6.33)	<0.0001 *	272 (227–353)	<0.001 *	10.25 (9.70–11.00)	<0.0001 *	6.14 (5.63–6.79)	<0.001 *
	Control	2.87 (2.53–3.58)		224 (212–257)		8.70 (8.20–9.10)		5.21 (4.70–5.45)	
**Location**	Right side	5.03 (4.19–5.40	0.2230	270 (208–351)	0.5459	10.27 (10.00–11.10)	0.4512	6.41 (5.72–6.93)	0.1244
	Left side	5.85 (4.24–6.35)		271 (228–358)		10.30 (9.70–11.00)		5.87 (5.48–6.86)	
**Tumor Size**	<3 cm	5.32 (4.24–5.97)	0.6298	272 (232–351)	0.8533	10.30 (9.0–11.00)	0.8532	5.84 (5.38–6.87)	0.4192
	≥3 cm	5.60 (3.75–6.37)		286 (221–385)		10.05 (9.80–10.80)		6.20 (5.72–6.68)	
**Grade**	Grade 2	3.70 (3.65–4.00)	<0.0001 *	272 (244–351)	0.8254	10.37 (10.00–10.90)	0.8254	6.13 (5.62–6.86)	0.1574
	Grade 3	5.75 (4.56–6.34)		276 (227–356)		10.20 (9.70–11.00)		6.16 (5.97–6.13)	
**Histological Type**	*Adenocarcinoma*	5.25 (4.19–6.33)	0.6989	270 (227–351)	0.8494	10.10 (9.70–11.00)	0.1238	5.98 (5.54–6.71)	0.1013
	*Mucinous* *adenocarcinoma*	5.36 (4.55–6.34)		282 (227–355)		10.70 (10.25–11.20)		6.60 (6.34–6.92)	
**Depth of Tumor Invasion**	T2	5.21 (4.14–6.15)	0.4642	278 (224–350)	0.3362	10.27 (9.60–10.70)	0.2663	6.14 (5.55–6.67)	0.7002
**(pT)**	T3	5.32 (4.21–6.34)		297 (228–351)		10.25 (9.70–11.00)		6.27 (5.61–6.86)	
	T4	5.36 (5.31–5.38)		331 (269–426)		10.15 (9.90–10.50)		5.94 (5.82–7.26)	
**Lymph Node Metastasis**	N0	5.03 (3.75–5.98)	0.3721	261 (220–340)	0.01 *	10.40 (9.70–11.20)	0.3118	5.98 (5.45–6.60)	0.0506 *
**(pN)**	N1 + 2	5.55 (4.97–6.34)		302 (268–426)		10.10 (9.70–10.70)		6.34 (5.77–7.04)	
**Distant Metastasis (pM)**	M0	5.25 (4.19–5.98)	0.1486	269 (227–314)	0.002 *	10.25 (9.70–11.00)	0.6448	6.02 (5.56–6.60)	0.0059 *
	M1	5.94 (5.15–6.80)		434 (388–450)		10.35 (9.70–10.50)		7.12 (6.86–7.53)	
**Stage at Diagnosis**	I	4.56 (3.75–5.86)	0.01 *#	248 (221–346)	0.01 #	10.12 (9.60–11.00)	0.3260	5.91 (5.43–6.61)	0.0183 *#
**(TNM)**	II	5.21 (3.86–6.17)		253 (218–295)		10.05 (9.70–11.20)		5.86 (5.43–6.47)	
	III IV	5.78 (4.26–6.34) 5.94 (5.15–6.80)		279 (244–351) 434 (3.88–450)		10.20 (9.80–10.70) 10.25 (9.70–10.50)		6.17 (5.77–6.87) 7.12 (6.86–7.53)	

* statistically significant study vs. control group; # statistically significant stage I vs. stage IV.

**Table 3:** Diagnostic usefulness of the platelet parameters evaluation in differentiating colorectal cancer (CRC) patients from healthy individuals.

	Cut-Off	Youden Index	AUC ± SE	Se [%]	Sp [%]	PPV [%]	NPV [%]	ACC [%]	*p*-Value
CLEC-2 [ng/mL]	3.71	0.74	0.924 ± 0.044	88	86	94	75	87	*p* < 0.0001
PLT [×10^3^/µL]	268	0.48	0.745 ± 0.055	59	89	95	38	66	*p* < 0.0001
MPV [fl]	9.30	0.92	0.957 ± 0.021	92	100	100	78	94	*p* < 0.0001
VEGF [pg/mL]	5.48	0.66	0.829 ± 0.052	82	83	94	57	82	*p* < 0.0001

**Table 4:** ROC curve analysis comparing sCLEC-2 with CA19-9 and CEA using the roccomp test.

Marker	AUC	Std. Error	95% CI	Comparison	Chi2	*p*-Value
sCLEC-2	0.9383	0.0453	0.8495–1.0000	sCLEC-2 vs. CA19-9	2.36	0.1241
CA19-9 (U/mL)	0.8117	0.0716	0.6714–0.9520			
sCLEC-2	0.9554	0.0416	0.8739–1.0000	sCLEC-2 vs. CEA	0.05	0.8268
CEA (ng/mL)	0.9665	0.0345	0.8989–1.0000			

**Table 5:** Univariate logistic regression for VEGF, CLEC-2, platelet parameters (PLT, MPV), and WBC in patients with colorectal cancer.

Variable	β	OR	95%CI	*p*-Value
Univariate Logistic Regression Analysis				
VEGF [pg/mL]	1.431	4.181	1.865–9.375	0.001
CLEC-2 [ng/mL]	1.849	6.35	2.086–19.371	0.001
PLT [×10^3^/µL]	0.011	1.011	1.002–1.020	0.015
MPV [fl]	1.371	3.941	1.870–8.304	<0.001
WBC × 10^3^	0.331	1.392	1.008–1.921	0.044

β—regression coefficient on the logit scale; OR—odds ratio; 95% CI—95% confidence interval.

## Data Availability

The datasets generated during and/or analyzed during the current study are available from the corresponding author on reasonable request.

## Supplementary Materials

The following supporting information can be downloaded at: https://www.mdpi.com/article/10.3390/diagnostics16071004/s1, Table S1: Summary of internal bootstrap validation. Figure S1: ROC curve for sCLEC-2 with pointwise 95% bootstrap confidence bands (1000 replicates).
