# Supplementary material for: sCLEC-2 (Soluble C-Type Lectin-like Receptor 2) as a New Diagnostic Marker of Platelet Activation in Colorectal Cancer Patients—A Preliminary Study
Source: Diagnostics (Basel). 2026 Mar 26;16(7):1004. doi: 10.3390/diagnostics16071004 (PMC13074032; doi:10.3390/diagnostics16071004)
Supplement: Supplementary file 1 [file diagnostics-16-01004-s001.zip › diagnostics-4190002-supplementary.pdf]

Supplementary Statistical Validation

Table S1. Summary of internal bootstrap validation

Metric	Value
**Sample size (complete cases)**	89 (CRC=64, controls=25)
**AUC (apparent)**	0.924
**AUC 95% CI (bootstrap)**	0.822–0.991
**AUC (optimism-corrected)**	0.924
**Odds Ratio per 1 ng/mL (apparent)**	6.357
**95% CI (Wald)**	2.086–19.371
**95% CI (bootstrap)**	2.809–459.783
**H0: AUC = 0.90 (one-sided p)**	0.261
**Post-hoc power (AUC>0.90; α=0.05)**	0.157
**Bootstrap replicates**	1000

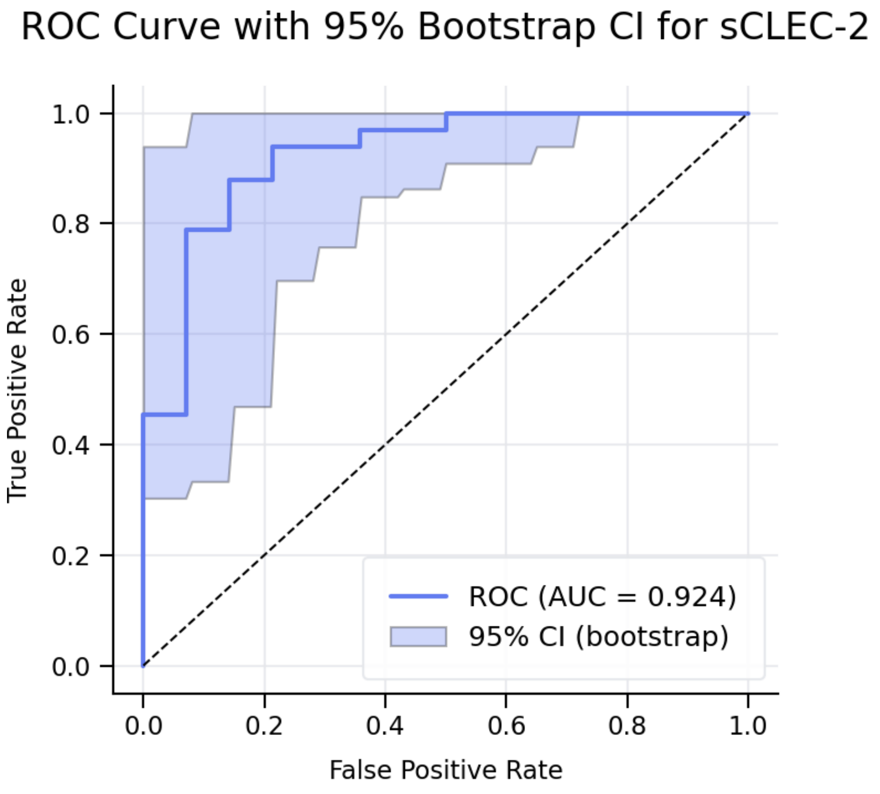

Figure S1. ROC curve for sCLEC-2 with pointwise 95% bootstrap confidence bands (1,000 replicates).
